# Evaluation of dose perturbations around iodine-125 seed sources in supplemental external beam prostate radiotherapy

**DOI:** 10.1093/jrr/rrad023

**Published:** 2023-05-05

**Authors:** Daisuke Kanda, Takashi Hanada, Kayo Yoshida, Tomoki Tanaka, Takahisa Eriguchi, Atsunori Yorozu, Toshio Ohashi, Naoyuki Shigematsu

**Affiliations:** Department of Radiology, Tokyo Medical Center, National Hospital Organization, Higashigaoka 2-5-1, Meguro-ku, Tokyo 152-8902, Japan; Department of Radiology, Tokyo Medical Center, National Hospital Organization, Higashigaoka 2-5-1, Meguro-ku, Tokyo 152-8902, Japan; Department of Radiology, Keio University School of Medicine, Shinanomachi 35, Shinjuku-ku, Tokyo 160-8582, Japan; Department of Radiology, Keio University School of Medicine, Shinanomachi 35, Shinjuku-ku, Tokyo 160-8582, Japan; Department of Radiology, Keio University School of Medicine, Shinanomachi 35, Shinjuku-ku, Tokyo 160-8582, Japan; Radiation Oncology Center, Ofuna Chuo Hospital, Ofuna 6-2-24, Kamakura, Kanagawa 247-0056, Japan; Department of Radiology, Tokyo Medical Center, National Hospital Organization, Higashigaoka 2-5-1, Meguro-ku, Tokyo 152-8902, Japan; Department of Radiology, Keio University School of Medicine, Shinanomachi 35, Shinjuku-ku, Tokyo 160-8582, Japan; Department of Radiology, Keio University School of Medicine, Shinanomachi 35, Shinjuku-ku, Tokyo 160-8582, Japan

**Keywords:** dose perturbation, iodine-125, brachytherapy (BT), external beam radiotherapy (EBRT)

## Abstract

We investigated dose perturbations caused by ^125^I seeds in patients undergoing supplemental external beam radiotherapy (EBRT) for prostate cancer. We examined two types of nonradioactive seed models: model 6711 and model STM1251. All experiments were performed using a water-equivalent phantom. Radiochromic film was used to measure the dose distributions adjacent to the seeds upstream and downstream of the external beam source. Single and clusters of multiple seeds were placed in slots in a solid water (SW) slab to measure dose perturbations with separate versus dense seed placement at beam energies of 6 or 10 MV. Monte Carlo simulations (MCSs) were also performed to include the theoretical basis against film dosimetry. Distinct patterns of dose enhancement (buildup [BU]) were upstream, and dose reduction (builddown [BD]) were downstream of the radiation source. Model 6711 with lower photon beam energies produced larger dose perturbations of BU and BD than the model STM1251. The results showed the same tendency with different seed placements and beam energies. However, these differences were not observed in the rotational irradiation measurement, which replicated a clinical plan. Dose perturbations around seeds result in dose enhancement and dose reduction with varying impact depending on the photon beam energy and seed type. This has the potential to cancel out these perturbations using multiple beam direction fields.

## INTRODUCTION

Radical prostatectomy, radiotherapy and hormonal therapy are standard therapeutic methods for prostate cancer. These methods are selected by considering the stage of malignancy [1, 2], as well as patient and physician preferences. External beam radiotherapy (EBRT) using a linear accelerator (LINAC) and brachytherapy (BT) using radioactive sources are the most common radiotherapy techniques. BT using low dose rate ^125^I seeds as a monotherapy is often performed for low-risk diseases. Selected intermediate-to-high-risk patients are often candidates for a combination of BT and EBRT, which is the recommended technique of the American Brachytherapy Society and National Comprehensive Cancer Network treatment guidelines [1–3].

For combined BT using ^125^I seeds and EBRT, there is no recommendation or evidence of advantages associated with administering EBRT before or after BT [3]. EBRT used as supplemental irradiation after BT can be used to adjust the dose distribution delivered to the prostate by BT. In addition, implanted ^125^I seeds in the prostate can be used as markers in image-guided radiotherapy during supplemental EBRT. In these cases, dose calculations for EBRT treatment planning are conducted by including ^125^I seeds, which are made of high atomic number (*Z*) materials such as silver and gold to be used as X-ray markers [4, 5].

Dose perturbations caused by high-Z materials have been reported [6–13]. To examine the effects of BT using ^125^I seeds, Steinman *et al.* examined the dose perturbation around a nonradioactive single seed using 6- and 18-MV photon beams and found that the dose perturbation was spatially limited to approximately 2 mm upstream and 5 mm downstream of the photon beam [6]. Since 40 to >100 seeds are used in actual practice, data for multiple seeds would be more informative.

The purpose of this study was to measure the dosimetric perturbations caused by the presence of a dense cluster of ^125^I seeds during the treatment of prostate cancer with supplemental EBRT. We examined two types of seeds to elucidate the magnitude of the dose perturbation under the condition of dense seed distribution and differing atomic numbers.

## MATERIALS AND METHODS

### Seed models

We used two types of ^125^I seed models available in Japan at the time of study: a cylindrical titanium capsule with a silver wire coated with a radioactive layer made of Ag^125^I and AgBr (model 6711, Oncura/Amersham, Buckinghamshire, UK) [4] and a cylindrical titanium capsule with a gold core rod and outer aluminum cylinder coated with thin layers of copper, nickel, and ^125^I (model STM1251, Bard Brachytherapy, Carol Stream IL, USA) [5]. We used non-radioactive seeds [14].

### Measurements

**Fig. 1:**
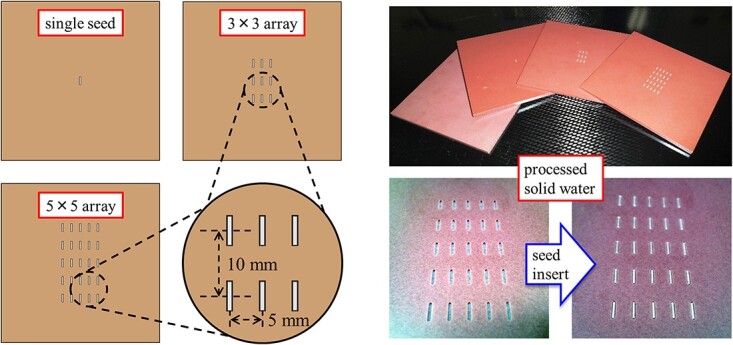
Schematic diagram and photograph of single, 3 × 3 and 5 × 5 ^125^I seed placement conditions.

All measurements were performed in a water-equivalent phantom (RT-3000-New, R-Tech, Tokyo, Japan) with a custom-designed 5 mm slab of solid water (SW) (Solid Water, Gammex RMI, Middleton WI, USA) to facilitate reproducible positioning and irradiation conditions for the two seed distributions. The slab was processed such that it contained a slot that allowed the placement of a single seed, a 3 × 3 array, or a 5 × 5 array of slots to accommodate nine or 25 seeds (Fig. 1).

**Fig. 2:**
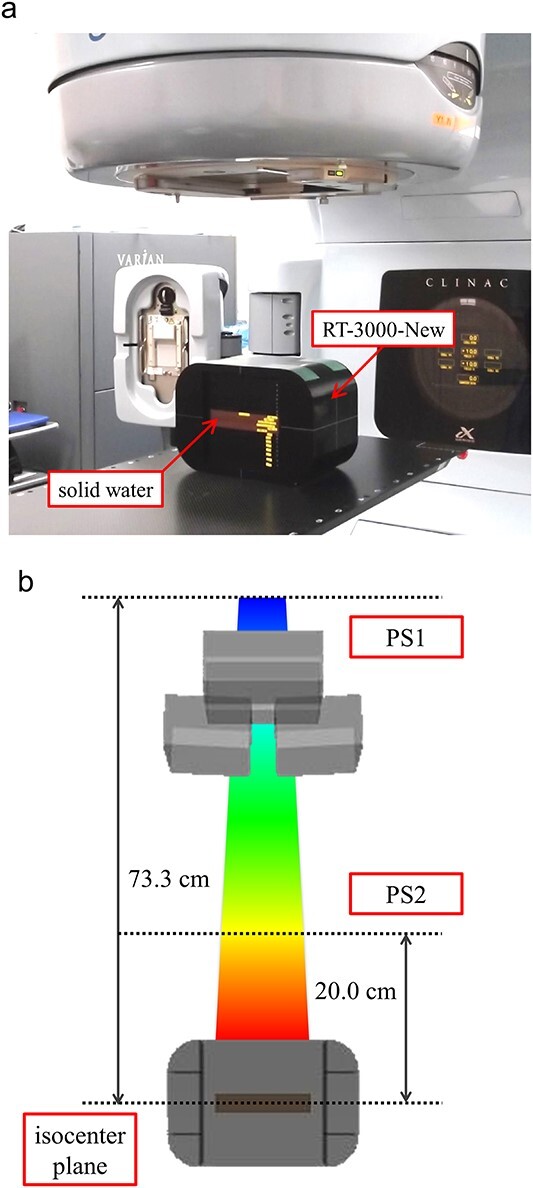
Photograph of the experimental setup (a) and schematic diagram of MCS (b) in this study.

**Fig. 3:**
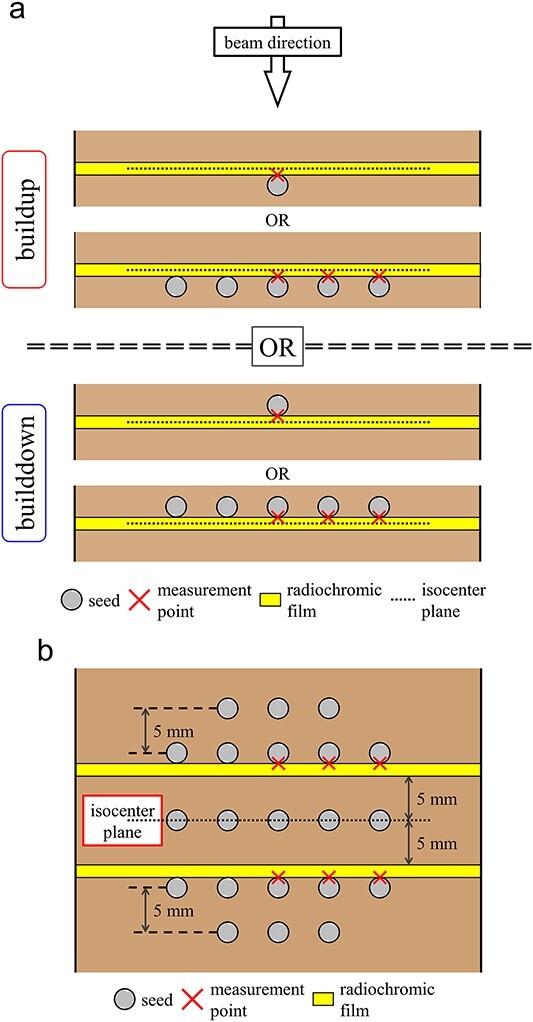
Schematic diagram of single and cluster seed placement conditions for upstream and downstream (a) dose perturbations in simple irradiation conditions and rotational irradiation measurements (b).

To elucidate the magnitude of the dose perturbation under simple irradiation conditions, a LINAC (CLINAC iX, Varian Medical Systems, Palo Alto, CA, USA) was used to deliver 2 Gy to the isocenter, 10 × 10 cm^2^ field size, single field irradiation with photon beam energies of 6 MV and 10 MV (Fig. 2a). Radiochromic film (EBT3, Ashland Inc., Wayne, NJ) was placed in a close contact with seeds at locations either upstream or downstream of the external beam source for each seed condition and on planes orthogonal to the radiation beam to obtain a 2D dose distribution (Fig. 3a). Irradiation of the seed placement of a single and 5 × 5 array was performed, and the film was placed in the isocenter position at a depth of 10 cm in the phantom for all measurements. In an actual patient using 3D conformal radiotherapy (3DCRT), intensity modulated radiotherapy (IMRT) or volumetric modulated arc therapy (VMAT) method of dose delivery, multiple seeds placed in a 3D arrangement were irradiated by multiple beam direction fields. Therefore, the dose perturbation in fixed field size (10 × 10 cm^2^) rotational irradiation with 3D seed placement was also investigated. The films were placed 5 mm upstream and downstream from the isocenter position, and 93 model STM1251 seeds were placed in a 3D arrangement using a slab combination of 3 × 3 and 5 × 5 arrays (Fig. 3b). A 10 MV photon energy was used, and the field size and delivery dose to the isocenter were the same as the simple irradiation condition measurements. Contents of experimental conditions are summarized in Table 1.

**Table 1:** Experimental conditions

Source model	6711 (Oncura/Amersham)		STM1251 (Bard Brachytherapy)		
Source array dimensional	One	Two	One	Two	Three
Number of seeds	1	25	1	25	93
Irradiation technique	Single field				Rotation
Field size	10 cm × 10 cm				
Beam energy (MV)	6, 10			10	

### Analysis method

The exposed films were digitized using a flatbed scanner (ES-10000G, Epson, Nagano, Japan) approximately 24 h after exposure. We obtained exposed film images in transmission mode with no filters or image-enhancement options, and with a resolution of 150 dpi. Data from the red channel were chosen for analysis due to high film sensitivity. We assumed that scan nonuniformity was present in both vertical direction (where vertical is the scanner lamp movement axis) and horizontal direction (where the horizontal axis is perpendicular to the scanner lamp movement axis). To minimize this nonuniformity as well as the film nonuniformity, pixel data signals were consistently averaged against the vertical (Y-axis referred as in-plane) and horizontal (X-axis referred as cross-plane) axes in the center of the irradiated field because all of the examined dose distributions of seeds and irradiated fields were symmetric in a 10 × 10-cm^2^ field along the X/Y-axes.

Reference measurements were obtained using an SW slab without seeds. To examine the difference in dose distribution with seeds relative to the reference dose distribution without seeds, the doses measured for each film were plotted according to the distance from the source center. This was normalized to the flat area and defined as the 4–6 cm outer area from the central field position. For an arbitrary position (*x*, *y*), the radiation doses without seeds $({D}_{x,y}^{\mathbf{ref}})$ and after installing the seeds $({D}_{x,y}^{\mathbf{seed}})$ were measured. The changes in dose $(\varDelta{D}_{x,y})$ for ${D}_{x,y}^{\mathbf{ref}}$ and ${D}_{x,y}^{\mathbf{seed}}$ were evaluated using the following equation: 


(1) \begin{equation*} \varDelta{D}_{x,y}\left(\%\right)=\frac{D_{x,y}^{\mathrm{seed}}-{D}_{x,y}^{\mathrm{ref}}}{D_{x,y}^{\mathrm{ref}}}\times 100 \end{equation*}

The $\varDelta{D}_{x,y}$ were reconstructed to 0.1 mm binning size and X-axis (cross-plane) dose profiles were obtained as a detailed result of the dose perturbation. These dose profiles were approximated as a linear combination of a Gaussian function and a Lorentzian function. This approximate function $F(x)$ therefore, is defined as:


(2) \begin{equation*} F(x)={F}_{0,\mathrm{G}}\pm \left(1-M\right)\cdot{H}_{\mathrm{G}}\cdot{e}^{-{\left(\frac{x-\mu }{W_{\mathrm{G}}}\right)}^2\cdot 4\cdot \ln 2}+{F}_{0,\mathrm{L}}\pm M\cdot \frac{H_{\mathrm{L}}}{4\cdot{\left(\frac{x-\mu }{W_{\mathrm{L}}}\right)}^2+1} \end{equation*}

where index $\mathrm{G}$ and $\mathrm{L}$ stand for Gaussian and Lorentzian, other ${F}_0$, $M$, $H$, $W$ and $\mu$ present offset level, component ratio of function, peak height, full width at half maximum (FWHM) and peak position, respectively. The $F(x)$ was optimized using generalized reduced gradient method to match the reference data with the dose perturbation for a ± 2.5 mm range from peak position.

### Monte Carlo simulations

Monte Carlo simulations (MCS) using Geant4 toolkit [15] version 10.07 patch 02 were also performed to include the theoretical basis against film dosimetry (Fig. 2b). The LINAC head geometry including X/Y collimator jaws was built and the photon beams were generated using phase space (PS1) files which are provided from the vendor (Varian Medical systems, Palo Alto, CA) that contain the particles at the entrance of the LINAC head (before the X/Y collimator jaws). In this study, Geant4 standard electromagnetic_opt3 physics package was employed in transport calculations.

The MCS was split into two stages. The first one tracks in the elements of the head the particles that come from a plane. This plane is considered as PS1, results of a Geant4 simulation of the first part of the LINAC (including the monitor chambers and flattening filter). A total of 50 PS1 files were used and 1 × 10^8^ primary particles for each data file, resulting in a total of 5 × 10^9^ primary particles were generated and tracked. As soon as the particles reached a plane that is 20 cm upstream from the isocenter in beam line (Z-axis), they were stored as new phase space (PS2) files. These PS2 files were used for a second simulation with the phantom setup and about 2 × 10^9^ primary particles were generated and tracked; the dose output data were retrieved and processed to obtain the final energy deposit distribution with a scoring resolution of 0.1 mm × 0.5 mm × 0.5 mm. All details of the MCS are summarized in Table 2 according to the recommendations of the American Association of Physicists in Medicine task group 268 (AAPM TG-268) report [16].

**Table 2:** Detailed information of the MCS according to the AAPM TG-268 report

Code, version, release date	Geant4 version 10.7 patch02 (June 11. 2021)
Validation	Comparisons to measured data and theory
Source	Phase-space file provided by Varian Medical Systems
Timing	21–38 hours on a 12 Intel® CoreTM i7-4960X nodes from 3.60 GHz
	11–21 hours on a 24 Intel® Xeon® CPU E5-2620 v3 nodes from 2.40 GHz
Physics and radiation transport parameters	
Physics List	G4EmStandardPhysics_option3
Photons	
Rayleigh scattering	Livermore models
Photoelectric effect	Livermore models
Compton scattering	Klein-Nishina model
e^−^/e^+^ pair production	BetheHeitler model with the LPM effect at high energies
Electrons and positrons	
Ionization model	Moller-Bhabha formulation
Multiple scattering	Urban model
Multiple scattering step limitation	UseDistanceToBoundary
Bremsstrahlung	eBremSB model and the eBremLPM model
Positron annihilation	eplus2gg model
Step size limitation (e−/e+)	Stepping function parameters: dRoverRange (0.2) and finalRange (0.1 mm)
Production cut	0.05 mm
Fluorescence	Activated
Variance reduction	No use
Scoring	Energy deposit in sensitive score region
Uncertainty calculation method	History-by-history method
Post-processing	Median filter was used before the analysis

CPU: Central processing unit; MCS: Monte Carlo simulations; AAPM TG-268: American Association of Physicists in Medicine task group 268; LPM: Landau–Pomeranchuk–Migdal

Percent depth dose of 6 MV and 10 MV beam in 10 × 10 cm^2^ field size with source-to-surface distance of 100 cm were calculated and compared for validation of LINAC model, achieving within 1.0% of the measured profiles within the 2–30 cm depth with a scoring resolution of 3 mm × 3 mm × 3 mm.

The seed models and RT-3000-New with SW were depicted truly and constructed in complete three-dimensions. The dimensions of seed models are the same as the ones used in the previous study [17–19]. The data from MCS were analyzed with same processes that were followed at film dosimetry.

## RESULTS

**Fig. 4:**
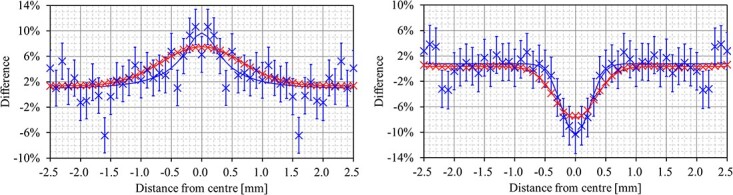
Example of the X-axis dose profile of the BU (left) and BD (right) for film dosimetry (red) and MCS (blue) using a photon beam energy of 10 MV for seed model STM1251. Note that profiles are symmetrical because pixel data signals were consistently averaged against the X/Y axes in the center of the irradiated field. Obtained raw data (×) and fitting function (solid line) are presented. Error bars are inserted in MCS results.

Figure 4 shows the X-axis dose profile with fitting function $F(x)$ as a detailed result of the dose perturbation observed with single seed location when model STM1251 nonradioactive seeds and 10 MV photon beam with single field were used. Note that the profiles were symmetrical because pixel data signals were consistently averaged against the X/Y-axes in the center of the irradiated field. As shown in Fig. 4, distinct patterns of increased dose representing BU dose were present upstream of the external beam source, and patterns of decreased dose representing BD dose were present downstream of the external beam source where the photon beam passed through the seeds.

Table 3 and 4 show the relative dose differences using single seed and 5 × 5 seed arrays placement with single field irradiation. Analysis of the dose perturbation with respect to differences in seed models showed larger impact on both BU and BD with model 6711 compared to model STM1251. Dose perturbation with respect to differences in beam energy showed greater dose change with 6 MV compared to 10 MV, with the exception of BU with STM1251 in single seed placement. The peaks of dose perturbation appear to be wider in BU from the results of FWHM values compared to BD. A comparison of the differences in seed models and beam energies indicates that the impact of dose perturbations has the same tendency under both dense and single-seed placement conditions. These tendencies yielded same results under both film dosimetry and MCS, with the results obtained from MCS had larger impact on dose perturbations on both BU and BD compared to film dosimetry. However, these differences, such as BU and BD, were not observed during rotational irradiation despite placing multiple seeds in a 3D arrangement, resulting in a mean dose change of 1.4–1.6% in film dosimetry and 2.2–2.5% in MCS at the position measured and is illustrated in Fig. 3b.

**Table 3:** Relative dose difference for the placement of single seed

Beam energy (MV)	Perturbation	Seed model	Method	Dose difference (%)	FWHM (mm)
6	Buildup	STM1251	Film	7.1	1.7
			MCS	12.9	1.0
		6711	Film	9.2	1.8
			MCS	14.9	1.2
	Builddown	STM1251	Film	-11.3	0.8
			MCS	-16.9	0.8
		6711	Film	-15.1	0.7
			MCS	-22.5	0.7
10	Buildup	STM1251	Film	7.5	1.8
			MCS	9.7	0.9
		6711	Film	7.7	1.9
			MCS	13.5	1.0
	Builddown	STM1251	Film	-7.8	0.8
			MCS	-10.5	0.5
		6711	Film	-11.2	0.7
			MCS	-17.3	0.5

MCS: Monte Carlo simulations; FWHM: full width at half maximum

**Table 4:** Relative dose difference for the placement of 5 × 5 seeds

Beam energy (MV)	Perturbation	Seed model	Method	Dose difference		FWHM	
				Mean (%)	S.D. (%)	Mean (mm)	S.D. (mm)
6	Buildup	STM1251	Film	6.2	1.2	1.7	0.2
			MCS	10.0	2.2	1.5	0.4
		6711	Film	9.5	0.9	1.8	0.1
			MCS	12.9	1.6	1.4	0.3
	Builddown	STM1251	Film	−10.9	0.7	0.8	0.1
			MCS	−13.8	1.6	0.7	0.1
		6711	Film	−15.0	0.7	0.8	0.0
			MCS	−19.3	1.4	0.6	0.1
10	Buildup	STM1251	Film	5.5	0.6	1.8	0.2
			MCS	9.1	0.8	1.4	0.3
		6711	Film	7.2	0.6	2.2	0.2
			MCS	12.4	1.5	1.3	0.3
	Builddown	STM1251	Film	−7.7	1.3	0.7	0.1
			MCS	−11.1	1.2	0.6	0.1
		6711	Film	−11.4	1.1	0.7	0.1
			MCS	−17.1	1.2	0.6	0.0

FWHM: full width at half maximum; MCS: Monte Carlo simulations; S.D.: standard deviation

## DISCUSSION

Various radiation interaction processes depend on the *Z* of the materials used. In the photon interaction cross section, both the probabilities of pair production and Bremsstrahlung interactions were proportional to *Z*^2^. For photoelectric interactions, the probability is approximately *Z*^3-5^ [20]. However, scattering photons interacting with materials that contribute to the dose perturbations in BU and BD can be considered negligible at the energies used in this study [12]. The behavior of secondary electrons arising from photon interactions with materials is the main factor affecting BU and BD [21]. The dose enhancement in BU mainly occurs because of the backscattering of secondary electrons arising from photon interactions with materials. In contrast, the dose reduction in BD mainly occurs due to the attenuation of secondary electrons by the X-ray markers made of high-Z materials inside the seed capsule. Our study showed that when comparing different seed models, dose perturbations in BU and BD were dependent on the materials in the seed. This was not unexpected because materials with a high *Z* were known to cause more backscattering and attenuation of secondary electrons [22, 23]. Dose perturbations should therefore theoretically have a greater impact in model STM1251, which used the higher *Z* material Au (*Z* = 79) as an X-ray marker than model 6711, which uses Ag (*Z* = 47). However, we observed the opposite effect wherein there was greater dose perturbations in model 6711 than in STM1251. We hypothesized that this discrepancy was due to the thicker metal used in model 6711 (0.5 mm in diameter), which may have caused more photon interactions and more scatter compared to model STM1251 (0.18 mm in diameter). The thickness difference in X-ray markers may be a greater factor affecting BU and BD than *Z* in this study. Similar data have been reported in an experimental study of esophageal stents [9]. As seen in Fig. 4 and results from FWHM values, dose perturbations in BU showed more dose spreading along the transverse axis compared to those in BD. This was due to upstream and downstream effects from different processes; that is, backscattered secondary electrons had a wider angular distribution than attenuated secondary electrons.

The dose perturbations in BU and BD were larger at 6 MV than at 10 MV beam energies. This suggests that as the incident photon beam energy was increased, there was less scatter and attenuation of secondary electrons, resulting in reduced dose perturbation from the ^125^I seeds. As previously mentioned, secondary electrons from interactions with incident photons influenced the BU and BD. The kinetic energy of the secondary electrons was proportional to the incident photon beam energy [20]. The range and transfer direction of the secondary electrons depend on the incident photon energy. Because the range of secondary electrons was shorter with photon beam energies of 6 MV compared to 10 MV, the rate of energy deposition around the seeds increased under the former condition. This result was similar to another study [6].

To replicate the clinical plan, rotational irradiation of multiple seeds placed in a 3D arrangement was performed. Our study showed that small dose perturbations occurred in either BU or BD. We speculated that BU and BD were canceled out by the multiple beam direction fields; therefore, the result would be useful information on BT and EBRT combination therapy. Although rotational irradiation techniques are not used clinically, it is important to see how the perturbation may cancel out in this scenario. However, further validation using a technique which replicates the clinical situation in a better manner, such as 3DCRT and/or IMRT techniques, for EBRT is required.

The diameter of X-ray markers made of high-Z materials inside the seed capsule are small (less than 1 mm) and external photon beam energies are relatively high (6 MV or 10 MV). Therefore, the seed model dependency on the dose perturbation should be detected with high dosimetry resolutions. In this study, dosimetry resolutions at X-axis profile were 0.17 mm (150 dpi) in film dosimetry and 0.1 mm in MCS, that were enough to detect the impact. Because of high dosimetry resolution, uncertainty in each method should be estimated. The typical values for uncertainties in radiochromic film dosimetry are reported from AAPM [24]. In general, the uncertainty of film uniformity is reported as 1.5% for currently available films. A typical value for uncertainty in scanner properties is reported as 0.5%. The uncertainty in selection of empirical fitting function of the calibration curve and fitting parameters is reported as 1.5%. The overall uncertainty could be expressed as relative standard uncertainties in quadrature, i.e. taking the square root of the sum of the squares. For the above values, overall uncertainty in film dosimetry was estimated as 2.2% (*k* = 1). If the uncertainty component of absorbed dose calibration of the LINAC beam (1.5%) is included, the above value will rise to 2.6% (*k* = 1) [25, 26]. Moreover, the measurement uncertainty was estimated from the standard error between the three exposed films, which were irradiated with reference conditions $({D}_{x,y}^{\mathrm{ref}})$. The uncertainties in several different areas (0.05–3.68 mm^2^) were approximately 1.9–2.1%. The type A uncertainty in MCS was estimated at approximately 3% at scoring resolution of 0.1 mm × 0.5 mm × 0.5 mm in this study. The MCS is based on a stochastic approach, which makes it subject to statistical uncertainty. The uncertainties per scoring voxels depend on simulation statistics (total number of primary particles) and the spatial resolution of scoring. The voxel size scored in this study was smaller than the calculation grid size in general treatment planning systems. The 1–2-mm grid size is often used in clinical settings. We devised the simulation to earn the particles that deposit the energies to these scoring volumes by splitting the simulation stages. Furthermore, analysis from the film dosimetry showed that an overall uncertainty of measurement chain of around 2–3% was estimated. Therefore, a statistical uncertainty of 3% on the MCS is considered non-problematic and relatively well done for sub-millimeter scoring voxel size aim to photon therapeutic beam energies [27]. Furthermore, the fitting function of Gaussian–Lorentzian was adopted to the peak and FWHM analyses due to the dose perturbation of BU and BD. This process was to find a mathematical function in an analytic form that best fits the set of data points to quantify a general trend and features in a data sequence. The results from peak fitting give the quantitative measure of robustness.

The results from film dosimetry and MCS indicated similar tendencies in dose perturbations, while the results from MCS showed larger impact than film dosimetry. This would be explained by the difference in dosimetry resolutions, since smaller resolution has smaller impact to partial volume effects. However, the most important fact is that the same tendencies focused on dose perturbations were shown between the two methods, and the dependencies on seed models and beam energies appeared. There was a similar result from another study, which concluded that the dose perturbations around the seed were investigated and depended on the seed type and beam energy [28].

**Fig. 5:**
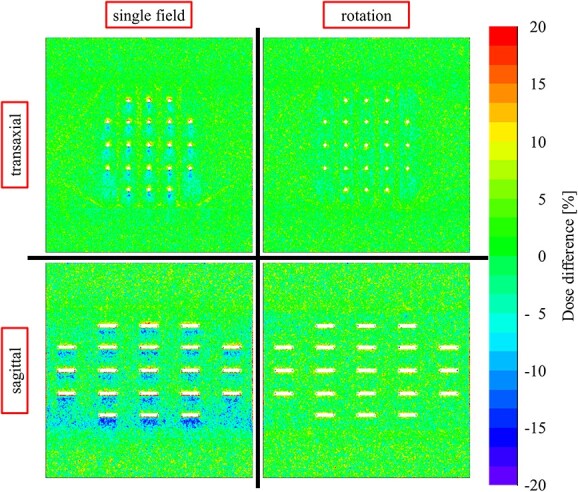
An example of the patterns of 2D dose perturbations observed at the central plane of the beam when the model STM1251 seeds and a photon beam of 10 MV are delivered by single field and rotational irradiation by MCS.

This study has some limitations. We only examined multiple seed placements in a single plane in a close contact state. A previous study reported that the dose perturbation was spatially limited to approximately 2 mm upstream and approximately 5 mm downstream of the incident beam [7]. In general, seeds were adequately spaced (5 mm) in a typical prostate ^125^I seed BT. Because dose perturbations around the seed were only observed approximately 5 mm from the seed, no additive perturbation effects should be observed in a BT implant. Therefore, we consider our study data to be broadly applicable to supplemental EBRT after ^125^I seed BT. In addition, we obtained an example of the patterns of 2D dose perturbations observed at the central plane of the beam when the model STM1251 seeds were used and a photon beam of 10 MV was delivered by single field and rotational irradiation by MCS (Fig. 5). From the Fig. 5, the dose perturbations apart from the examined area also seemed to be suppressed and were cancelled out using multiple beam direction fields compared to a single field, indicating that irradiation techniques used in clinical situations, such as 3DCRT, IMRT and rotational IMRT methods of dose delivery, consisting of multiple beam direction fields, may aid in suppression of the dose perturbations due to seed insertions.

We investigated the magnitude of dose perturbations under single and multiple seed placement conditions. These dose perturbations resulted in dose enhancement and reduction around the seeds, with varying impacts depending on the photon beam energy and seed type. In addition, it showed the same tendency in single and dense seed placement conditions under simple irradiation conditions, but not in the rotational irradiation measurement. These results provided useful information regarding BT and EBRT combination therapy.

## CONFLICT OF INTEREST

The authors have no conflicts of interest to declare.

## PRESENTATION AT A CONFERENCE

Poster presentation at the 35th annual meeting of the Japanese Society for Radiation Oncology (JASTRO).

## FUNDING

Part of this work was supported by the Japan Society for the Promotion of Science (JSPS) KAKENHI Grant Number JP16K19849 and JP21K07685.

## DATA AVAILABILITY

The data related to this article will be shared on reasonable request to the corresponding author.
